# Isolation of a Gamma‐Aminobutyric Acid (GABA)‐Producing *Lactiplantibacillus plantarum* XBMU‐SN‐23 and Optimization of Its Culture Medium Composition and Fermentation Conditions

**DOI:** 10.1002/fsn3.71782

**Published:** 2026-04-13

**Authors:** Dandan Gao, Chenchen Wang, Jiwen Wang, Junyao Mei, Yujie Cao, Xuankang Yang, Jinpu Ma, Shangyun Kang, Jinyong Ma, Ping Li

**Affiliations:** ^1^ Key Laboratory of Biotechnology and Bioengineering of State Ethnic Affairs Commission, Biomedical Research Center Northwest Minzu University Lanzhou China; ^2^ College of Life Sciences and Engineering Northwest Minzu University Lanzhou China; ^3^ Gansu Puluo Biotech Co., Ltd. Yumen China; ^4^ Key Laboratory for Food Microbiology and Nutrition of Zhejiang Province, School of Food Science and Biotechnology Zhejiang Gongshang University Hangzhou China

**Keywords:** fermentation, GABA, *Lactiplantibacillus plantarum*, optimization, RSM

## Abstract

Gamma‐aminobutyric acid (GABA) is a crucial inhibitory neurotransmitter with diverse physiological functions, including the treatment of epilepsy, alleviation of anxiety, and improvement of depressive symptoms. Lactobacillus has emerged as an ideal genus for GABA biosynthesis due to its high safety, stable and controllable fermentation process, and broad application scope. In this study, a high‐yield GABA‐producing strain XBMU‐SN‐23 was isolated from Lanzhou Jiangshui and preliminarily identified as *Lactiplantibacillus plantarum* via 16S rDNA sequencing. The GABA yield of this strain under unoptimized conditions was 2.150 g/L. The Box–Behnken design combined with response surface methodology (RSM) was applied to optimize the medium components and fermentation conditions. The optimal medium composition was determined as: Tween 80 at 0.4% (v/v), Mg^2+^ at 0.1 mg/mL, and Ca^2+^ at 5 mg/mL, under which the actual GABA yield reached 2.618 g/L. The optimal fermentation parameters were an initial pH of 5, an inoculum size of 2.5%, and a fermentation time of 36 h, leading to an actual GABA yield of 3.562 g/L. After optimization, the GABA yield was increased by 65.7% compared with the unoptimized level. This study clarified the optimal culture conditions for high GABA production by *L. plantarum* XBMU‐SN‐23, laying a foundation for its potential application in GABA‐enriched fermented products.

## Graphical abstract

In this study, a high GABA‐producing strain was isolated and screened from Jiangshui, through isolation and purification, Gram staining, and HPLC determination. The strain was identified as *Lactiplantibacillus plantarum* XBMU‐SN‐23 via 16S rDNA gene sequence analysis. After systematically determining the physiological and growth characteristics of the strain, the BBD‐RSM was applied to optimize the culture medium components (Tween 80, MgSO_4_, MnSO_4_, CaCl_2_) and fermentation conditions (inoculum size, initial pH, fermentation time), respectively, which significantly enhanced the GABA production capacity of the strain.

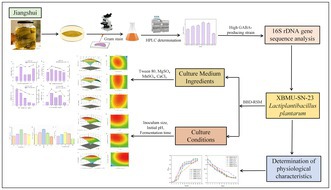

## 1 Introduction

GABA is a bioactive compound with diverse physiological functions that acts as a critical inhibitory neurotransmitter in the mammalian central nervous system (Kim and Yoon 2023). Specifically, its physiological roles encompass regulating blood pressure, improving brain function, and exerting sedative and anxiolytic effects, among others (Kalueff and Nutt 2007; Ajibola et al. 2021; Doyno and White 2021; Li, Pei, et al. 2023). These remarkable physiological activities endow GABA with broad application prospects and great development potential in various fields, such as food, medicine, and health care products (Zhang et al. 2024).

At present, GABA production can be broadly categorized into three primary methods: chemical synthesis, plant‐based enrichment, and microbial fermentation (Han et al. 2023). Although chemical synthesis enables large‐scale industrial production, it has a series of notable drawbacks, including difficulties in ensuring product safety and potential environmental pollution during the production process (Zhu et al. 2017). The plant‐based enrichment method produces GABA with relatively low content (Liao et al. 2017), which is insufficient to meet the growing market demand for this compound in practical applications. In contrast, microbial fermentation has become the primary research focus for GABA yield (Iorizzo et al. 2023). This method has attracted considerable attention from researchers due to its distinct advantages: low production cost, high product safety, and short production cycle.

Nowadays, the main strains utilized for the fermentative preparation of GABA include *Lactobacillus*, *Corynebacterium glutamicum* , *Bacillus subtilis* , and *Escherichia coli* (Wan‐Mohtar et al. 2020; Luo et al. 2021). Among these GABA‐producing strains, lactic acid bacteria are widely recognized as safe microorganisms, and they have been reported to synthesize larger amounts of GABA compared with other microorganisms (Kim et al. 2022). For example, Diez‐Gutiérrez et al. utilized *Lactiplantibacillus plantarum* K16 to produce GABA through fermentation to obtain 1 g/L GABA (Diez‐Gutiérrez et al. 2022). In addition, it can promote the synthesis of GABA and achieve higher yield efficiency by adjusting the composition of the culture medium, fermentation temperature, and pH value. Cai et al. investigated the optimization of the GABA yield by *L. plantarum* FRT7 and found that the maximum production reached 1.16 ± 0.02 g/L after 48 h of incubation at 40°C with an initial pH of 7.0 in MRS medium supplemented with 3% monosodium glutamate (MSG) and 2 mmol/L pyridoxal phosphate (PLP) (Cai et al. 2023).

RSM is an empirical statistical modeling technique used for multiple regression analysis. It employs quantitative data obtained from well‐designed experiments to simultaneously solve multiple equations (Ghellam et al. 2021). Designing experiments using RSM can explore the synergistic effects of multiple parameters through statistical methods, with the advantage of identifying nonlinear relationships among independent variables (El‐Mekkawi et al. 2019). Furthermore, a GABA yield prediction model enhances the application of this methodology. For example, Li et al. used *Levilactobacillus* sp. LB‐2 for GABA fermentation and optimized the yield conditions via RSM (37°C, pH 4.5, L‐MSG concentration 2.5 g/L). The maximal GABA yield achieved was 49.42 g/L, representing a 3.5‐fold increase compared with the previous yield level of 14.19 g/L (Li, Li, et al. 2023).

In this study, we aimed to isolate GABA‐producing bacteria and identified the strains using both morphological and molecular biological methods. Additionally, we optimized the composition of the culture medium for strain XBMU‐SN‐23 by RSM based on the BBD and investigated the optimal fermentation conditions to achieve high levels of GABA, focusing on inoculum size, initial pH, and fermentation time. These results may contribute to the development of GABA‐enriched functional foods.

## 2 Materials and Methods

### 2.1 Materials and Reagents

Lanzhou's old yogurt and Jiangshui were purchased from a specialty supermarket in Lanzhou (Gansu, China). Agar powder, MRS broth medium, and sodium l‐glutamate (≥ 98%) were obtained from Beijing Solarbio Technology Co. Ltd. (Beijing, China). Anhydrous ethanol was sourced from Tianjin Chemical Reagent Factory (Tianjin, China). Ninhydrin hydrate (≥ 98%) was acquired from Shanghai Yuanye Biotechnology Co. Ltd. (Shanghai, China). The GABA standard (≥ 99%) was purchased from Qingdao Jieshikang Biotechnology Co. Ltd. (Qingdao, China). Sodium chloride, ammonium sulfate, chromatographic‐grade methanol, chromatographic‐grade acetonitrile, and sodium acetate were obtained from Chemical Reagent Co. Ltd. (China). Dansyl chloride, pyridoxal phosphate, ammonium chloride, calcium chloride, and magnesium sulfate were sourced from Shanghai McLean Biochemical Technology Co. Ltd. (Shanghai, China). Peptone, beef extract, Tween 80, and yeast extract were purchased from Qingdao Haibo Biotechnology Co. Ltd. (Qingdao, China).

### 2.2 Screening and Isolation of GABA‐Producing Strains

0.5 mL of each sample was accurately pipetted into 4.5 mL of sterile saline, and the mixture was homogenized by shaking in a constant‐temperature shaking incubator (ZWYR‐2401, Shanghai Zhicheng Analytical Instrument Manufacturing Co. Ltd., Shanghai, China), followed by serial dilution to 10^−1^, 10^−2^, 10^−3^, and 10^−4^ gradients. Then, 200 μL of each dilution was spread on MRS solid medium, with three parallel replicates set for each dilution. After incubation at 37°C for 48 h in a constant‐temperature incubator (LRH: 150, Shanghai Yiheng Science and Technology Co. Ltd., Shanghai, China), single colonies with good morphology were selected for streak purification and incubated for another 48 h. The purified single colonies were labeled and inoculated into MRS liquid medium, and then followed by static cultivation at 37°C for 24 h. After 2–3 subcultures, the strains were subjected to smear preparation, Gram staining, and microscopic observation using a microscope (NE300, Shenzhen Sinico Optical Instrument Co. Ltd., Shenzhen, China) to confirm the acquisition of Gram‐positive pure cultures. The pre‐preserved strains were inoculated into MRS liquid medium and activated for two consecutive generations. Subsequently, the strains were inoculated at an inoculum size of 3% into fermentation medium supplemented with 1.5% l‐glutamic acid (as the substrate for GABA synthesis) and cultured at 37°C for 30 h. The supernatant collected after centrifugation was used for quantitative analysis by high‐performance liquid chromatography (HPLC; Model 1260, Agilent Technologies Co. Ltd., Beijing, China). For sample derivatization and analysis, 200 μL of the sample was mixed with 200 μL NaHCO_3_‐Na_2_CO_3_ buffer (pH 11) and 400 μL of 10 g/L dansyl chloride (DNS‐Cl) solution. After thorough vortexing, the mixture was incubated at 30°C for 20 min and filtered through a 0.45 μm organic filter membrane. Chromatographic separation was performed on a ZORBAX SB C18 column (4.6 mm × 250 mm, 5 μm). The mobile phase consisted of 0.025 mol/L sodium acetate (A) and acetonitrile (B). The flow rate was 1.0 mL/min, column temperature was 40°C, injection volume was 10 μL, and detection was performed at 340 nm.

### 2.3 Determination of GABA Content by Berthelot Method

According to the modified method of Huang et al. (2025) the activated XBMU‐SN‐23 strain was inoculated into 20 mL of liquid fermentation medium at an inoculum size of 1% (v/v). Following anaerobic incubation at 37°C for 48 h, cells were harvested by centrifugation at 10000 r/min for 10 min at 4°C, and the supernatant was discarded. Subsequently, the cell pellet was washed three times with phosphate‐buffered saline (PBS, pH 7.2), weighed using a precision balance (105‐AL, Mettler Bio‐equipment Co. Ltd., Shanghai, China), and then resuspended at a solid‐to‐liquid ratio of 1:10 (w/v) by adding PBS (pH 7.2) containing 0.2 mg/mL lysozyme, 0.2% β‐mercaptoethanol, and 0.1 mM PLP. The suspension was then incubated in a water bath at 37°C for 1 h. After incubation, the mixture was centrifuged at 4°C for 10 min to collect the supernatant, which was designated as the crude glutamate decarboxylase (GAD) enzyme solution.

GABA content is determined and GAD activity is calculated according to the method of Zhang et al. (2022) with slight modifications. GABA standard solutions were prepared at concentrations of 2, 4, 6, 8, and 10 g/L. After color development under the same treatment conditions as the samples, the absorbance was measured at 640 nm. A standard curve was established with GABA concentration as the X‐axis and absorbance as the *Y*‐axis. For sample determination, 0.5 mL of crude GAD enzyme solution was mixed with 0.2 mL of boric acid solution (pH 9.0, 0.2 mol/L), 1 mL of 6% (w/v) phenol solution, and 0.4 mL of 7% sodium hypochlorite solution. After thorough mixing, the mixture was incubated in a water bath at 98°C for 10 min for color development. The mixture was immediately cooled in an ice bath when a blue–green color appeared, and 2 mL of 60% (v/v) ethanol solution was then added. After shaking and homogenization, the absorbance was measured at 640 nm. GABA yield was calculated based on the standard curve, and GAD activity was indirectly determined according to the amount of GABA produced per unit time. One unit of enzyme activity (U) is defined as the amount of enzyme that catalyzes the formation of 1 μmol GABA per h (Ding et al. 2023).

### 2.4 Identification of XBM‐SN‐23 by 16S rDNA Gene Sequencing

The genomic DNA of the strain with the highest GABA yield was extracted using a bacterial DNA extraction kit (Sangon Biotech Co. Ltd., Shanghai, China), followed by PCR amplification of the 16S rDNA with universal primers. The universal primers used were: 27F (5′‐AGAGTTTGATCCTGGCTCAG‐3′) and 1492R (5′‐GGTTACCTTGTTACGACTT‐3′).

The 20 μL PCR reaction system consisted of the following components: 2.0 μL of template DNA, 0.4 μL of Taq DNA polymerase, 1.6 μL of Mg^2+^ solution, 2.0 μL of 10× PCR buffer, 2.0 μL of dNTP (2.5 mmol/L), 2.0 μL each of upstream and downstream primers (10 μmol/L), and 8.0 μL of ddH_2_O.

The PCR reaction conditions were as follows: pre‐denaturation at 94°C for 5 min, followed by 30 amplification cycles (denaturation at 94°C for 30 s, annealing at 58°C for 30 s, and extension at 72°C for 1 min), a final extension at 72°C for 10 min, and storage at 4°C. The PCR products were analyzed by 0.8% agarose gel electrophoresis (150 V, 100 mA, 20 min).

After electrophoresis, the gel containing the target fragments was excised, and the desired fragments were recovered and sequenced by Gansu Huake Biotechnology Co. Ltd. The sequencing results were aligned with sequences in the GenBank database of the National Center for Biotechnology Information (NCBI), and a phylogenetic tree was constructed.

### 2.5 Determination of Growth Capacity of Strain

The strains were inoculated into 10 mL of MRS liquid medium at an inoculum size of 2% (v/v) and incubated at 37°C for 24 h. Bacterial suspensions were collected at 2 h intervals. The absorbance of the samples was measured at 600 nm using a UV spectrophotometer (SPD15C, Mettler Bio‐equipment Co. Ltd., Shanghai, China). The growth capacity of the strains was evaluated with incubation time as the x‐axis.

### 2.6 Determination of Acid Production Capacity

The strains were inoculated into 10 mL of MRS liquid medium at a 2% inoculum size and incubated at a constant temperature of 37°C for 24 h. Bacterial suspension was collected every 2 h. The pH values of the strains were measured using a pH meter (PHS‐3E, Shanghai Yidian Scientific Instrument Co. Ltd., Shanghai, China) at various time points. The incubation time was used as the horizontal axis, and the pH values at different intervals were plotted to determine the acid‐producing capacity.

### 2.7 Optimization of the Culture Medium Ingredients for Strain XBMU‐SN‐23

#### 2.7.1 One Factor at a Time Method

The OFAT method was employed to determine the optimal fermentation medium ingredients for GABA yield by *L. plantarum* XBMU‐SN‐23. The optimized parameters included Tween 80 concentrations (0.2%, 0.4%, 0.6%, 0.8%, and 1.0%), MgSO_4_ concentrations (0, 0.1, 0.2, 0.3, and 0.4 mg/mL), MnSO_4_ concentrations (0, 0.02, 0.04, 0.06, and 0.08 mg/mL), and CaCl_2_ concentrations (0, 5, 10, 15, and 20 mg/mL). MRS medium (pH 4.8) was used as the basal medium for 24 h fermentation to investigate the effects of each factor on GAD activity and GABA yield. The accumulation of GABA in the medium was detected using the Berthelot method. Each experiment was repeated in triplicate.

#### 2.7.2 Response Surface Methodology

Through the analysis and treatment of OFAT experiment results and based on the *Box–Behnken* design (Table 1), a three‐factor, three‐level response surface analysis test was designed using Design‐Expert v8.0.6.1 with GABA content as the response value to determine the conditions for optimal GABA yield from strain *L. plantarum* XBMU‐SN‐23. The validity of the model was verified by comparing the model‐predicted values with the experimental values. The quadratic equation is as follows:

$$Y=K_{0}+\sum_{i=1}^{3}\;K_{i}X_{i}+\sum_{i=1}^{2}\;\sum_{j=i+1}^{3}\;K_{\mathrm{ij}}X_{i}X_{j}+\sum_{i=1}^{3}\;K_{\mathrm{ii}}X_{i}^{2}+e$$

*K*_0_ is constant; *K*_*i*_, *K*_*j*_, and *K*_*ii*_ are coefficients of variables, *X*_*i*_ and *X*_*j*_ indicate the levels of independent variables; *e* is experimental error.

**TABLE 1:** Factors and levels of *Box–Behnken* experiments for optimizing culture medium.

Level	Factor		
	*X*_1_‐Tween 80 (% v/v)	*X*_2_‐MgSO_4_ (mg/mL)	*X*_3_‐CaCl_2_ (mg/mL)
−1	0.2	0.0	0
0	0.4	0.1	5
1	0.6	0.2	10

### 2.8 Optimization of Culture Conditions for Strain XBMU‐SN‐23

#### 2.8.1 One Factor at a Time Method

In this study, *L. plantarum* XBMU‐SN‐23, which exhibited the highest GABA yield capacity, was selected for culture condition optimization using the one‐factor‐at‐a‐time (OFAT) method. In this approach, the fermentation temperature was fixed at 37°C, and only one factor was varied at a time while all other factors remained constant to assess its effect on GABA content. The parameters optimized included inoculum size (1.5%, 2.0%, 2.5%, 3.0%, and 3.5%), initial pH (4.0, 4.5, 5.0, 5.5, and 6.0), and fermentation times (12, 24, 36, 48, and 60 h). GABA accumulation in the fermentation broth was determined using the Berthelot method. All experiments were performed in triplicate.

#### 2.8.2 Response Surface Methodology

Based on the results of OFAT experiments, a three‐factor and three levels were used to optimize the fermentation conditions following the *Box–Behnken* design (Table 2). GABA content was used as the response value to determine the optimal conditions for GABA yield by *L. plantarum* XBMU‐SN‐23. Moreover, the model's validity was verified by comparing the model‐predicted values with experimental values. The quadratic equation is as follows:

$$y=b_{0}+\sum_{j=1}^{m}\;b_{j}x_{j}+\sum_{i\leq j=1}^{m}\;b_{\mathrm{ij}}x_{i}x_{j}+\sum_{i=1}^{m}\;b_{i}x_{i}^{2}+e$$

*b*_0_ is constant; *b*_*j*_, *b*_*ij*_, and *b*_*i*_ are coefficients of variables, *x*_*i*_ and *x*_*j*_ indicate the levels of independent variables, *e* is experimental error, *m* = 3.

**TABLE 2:** Factors and levels of *Box–Behnken* experiments for optimizing fermentation conditions.

Factor	Level		
	−1	0	+1
*x*_1_‐pH	4.5	5.0	5.5
*x*_2_‐inoculum size (%) v/v	2.0	2.5	3.0
*x*_3_‐fermentation time (h)	24	36	48

### 2.9 Statistical Analysis

All quantitative data in this study were expressed as mean ± standard deviation (SD) with three independent replicates (*n* = 3). Design‐Expert software (v8.0.6, Stat‐Ease Inc., Minneapolis, MN, USA) was used for RSM experimental design. Statistical analyses were conducted using SPSS software (v.19.0, Chicago, IL, USA), while Origin 2021b (OriginLab Inc., Northampton, MA, USA) was employed for data visualization. Furthermore, comparisons among multiple groups were performed using a one‐way ANOVA test, with a significance level set at *p* < 0.05.

## 3 Results

### 3.1 Isolation and Screening of GABA‐Producing Lactic Acid Bacteria

**FIGURE 1:**
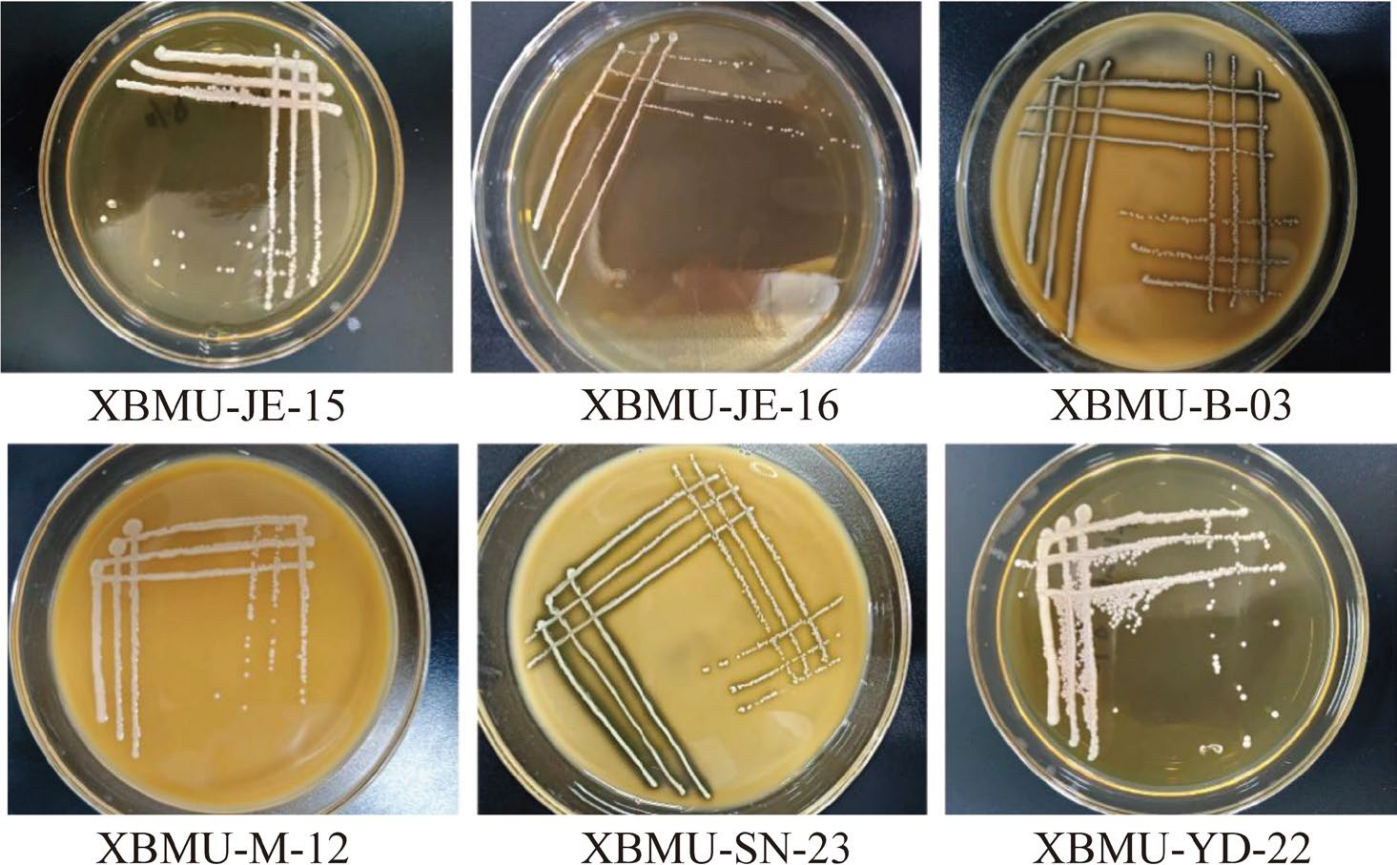
Morphological observation of GABA‐producing strains.

**FIGURE 2:**
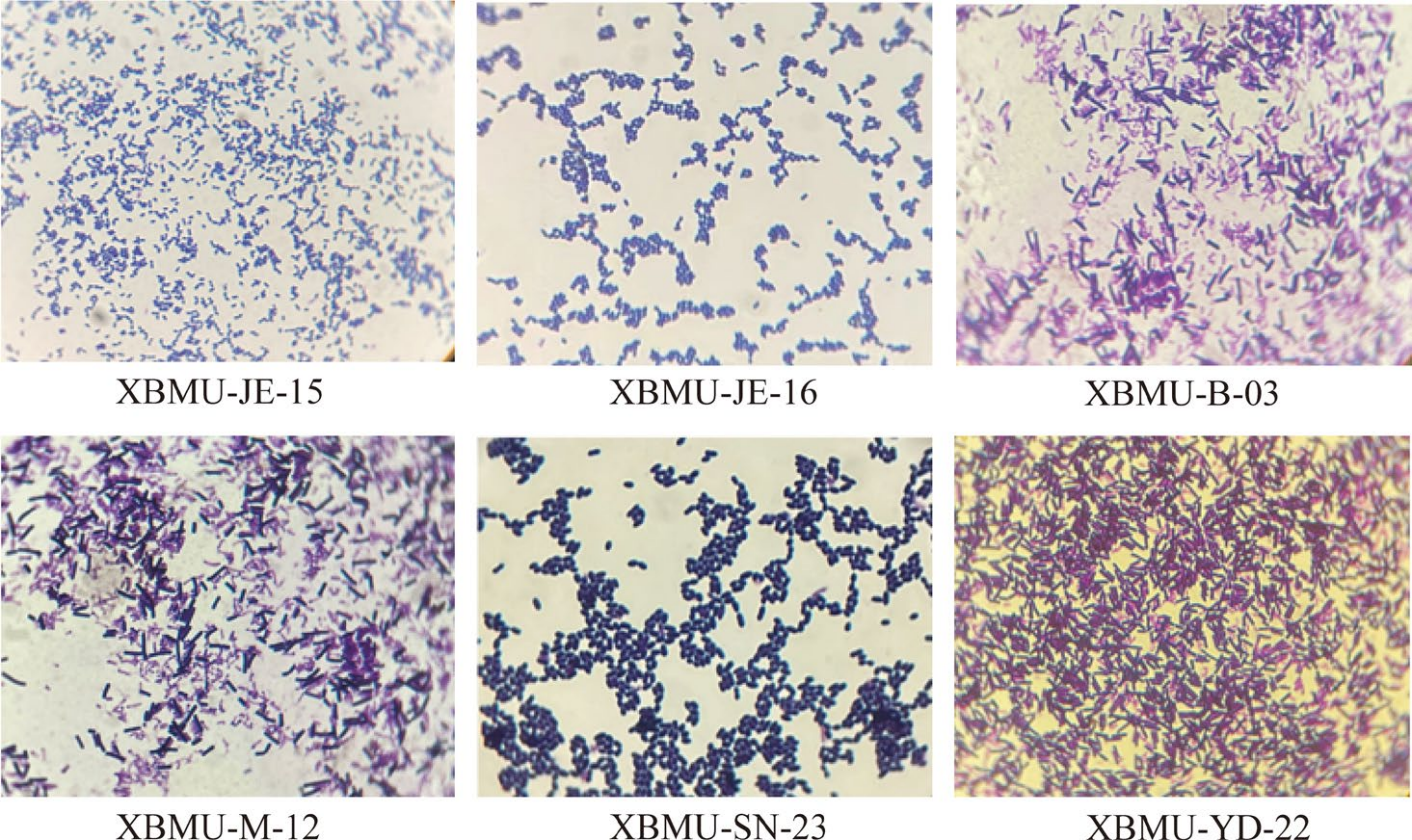
Gram stain results.

**FIGURE 3:**
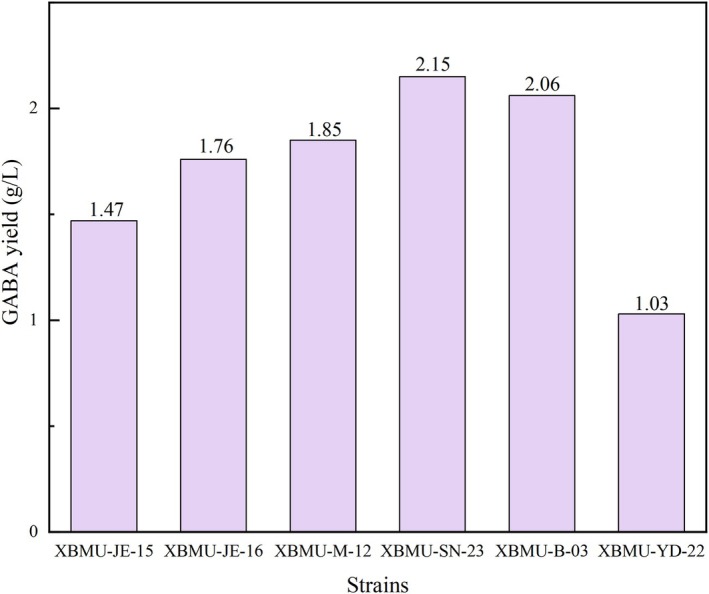
The ability of six strains to produce GABA.

Strains were cultured on MRS solid medium at 37°C for 48 h. Six GABA‐producing lactic acid bacterial strains were screened, and their colony morphologies were shown in Figure 1. The colonies appeared creamy white, with neat and smooth edges, a moist and shiny surface, and a raised, opaque center. Microscopic observation showed that the bacterial cells were Gram‐positive and appeared as rod‐shaped after crystal violet staining, as illustrated in Figure 2. Among the tested strains, XBMU‐SN‐23 exhibited the highest GABA yield capacity, achieving a yield of 2.150 g/L, which is shown in Figure 3.

### 3.2 Identification of XBMU‐SN‐23 by 16S rDNA Sequences

The DNA sequence of strain XBMU‐SN‐23 is shown in Table 3. The 16S rDNA gene sequence of XBMU‐SN‐23 was compared with sequences in the GenBank database. A phylogenetic tree was constructed using MEGA 7.0 software, revealing that strain XBMU‐SN‐23 shares 99% sequence similarity with *L. plantarum* K‐D513. Based on this high similarity and phylogenetic analysis, strain XBMU‐SN‐23 was preliminarily identified as *L. plantarum*.

**TABLE 3:** 16S rDNA sequence of strain XBMU‐SN‐23.

Serial number	Strain gene sequences
1–50	CGAACGCTGGCGGCGTGCCTAATACATGCAAGTCGAACGAACTCTGGTAT
51–100	TGATTGGTGCTTGCATCATGATTTACATTTGAGTGAGTGGCGAACTGGTG
101–150	AGTAACACGTGGGAAACCTGCCCAGAAGCGGGGGATAACACCTGGAAACA
151–200	GATGCTAATACCGCATAACAACTTGGACCGCATGGTCCGAGTTTGAAAGA
201–250	TGGCTTCGGCTATCACTTTTGGATGGTCCCGCGGCGTATTAGCTAGATGG
251–300	TGGGGTAACGGCTCACCATGGCAATGATACGTAGCCGACCTGAGAGGGTA
301–350	ATCGGCCACATTGGGACTGAGACACGGCCCAAACTCCTACGGGAGGCAGC
351–400	AGTAGGGAATCTTCCACAATGGACGAAAGTCTGATGGAGCAACGCCGCGT
401–450	GAGTGAAGAAGGGTTTCGGCTCGTAAAACTCTGTTGTTAAAGAAGAACAT
451–500	ATCTGAGAGTAACTGTTCAGGTATTGACGGTATTTAACCAGAAAGCCACG
501–550	GCTAACTACGTGCCAGCAGCCGCGGTAATACGTAGGTGGCAAGCGTTGTC
551–600	CGGATTTATTGGGCGTAAAGCGAGCGCAGGCGGTTTTTTAAGTCTGATGT
601–650	GAAAGCCTTCGGCTCAACCGAAGAAGTGCATCGGAAACTGGGAAACTTGA
651–700	GTGCAGAAGAGGACAGTGGAACTCCATGTGTAGCGGTGAAATGCGTAGAT
701–750	ATATGGAAGAACACCAGTGGCGAAGGCGGCTGTCTGGTCTGTAACTGACG
751–800	CTGAGGCTCGAAAGTATGGGTAGCAAACAGGATTAGATACCCTGGTAGTC
801–850	CATACCGTAAACGATGAATGCTAAGTGTTGGAGGGTTTCCGCCCTTCAGT
851–900	GCTGCAGCTAACGCATTAAGCATTCCGCCTGGGGAGTACGGCCGCAAGGC
901–950	TGAAACTCAAAGGAATTGACGGGGGCCCGCACAAGCGGTGGAGCATGTGG
951–1000	TTTAATTCGAAGCTACGCGAAGAACCTTACCAGGTCTTGACATACTATGC
1001–1050	AAATCTAAGAGATTAGACGTTCCCTTCGGGGACATGGATACAGGTGGTGC
1051–1100	ATGGTTGTCGTCAGCTCGTGTCGTGAGATGTTGGGTTAAGTCCCGCAACG
1101–1150	AGCGCAACCCTTATTATCAGTTGCCAGCATTAAGTTGGGCACTCTGGTGA
1151–1200	GACTGCCGGTGACAAACCGGAGGAAGGTGGGGATGACGTCAAATCATCAT
1201–1250	GCCCCTTATGACCTGGGCTACACACGTGCTACAATGGATGGTACAACGAG
1251–1300	TTGCGAACTCGCGAGAGTAAGCTAATCTCTTAAAGCCATTCTCAGTTCGG
1301–1350	ATTGTAGGCTGCAACTCGCTACATGAAGTCGGAATCGCTAGTAATCGCGG
1351–1400	ATCAGCATGTGCGGTGAATACGTTCCCGGGTCTTGTACACACCGCCCGTC
1401–1450	ACACCATGAGAGTTTGTAACACCCAAAGTCGGTGGGGTAACCTTTTAGGA
1451–1454	ACCA

### 3.3 Growth Capacity of Strain XBMU‐SN‐23

**FIGURE 4:**
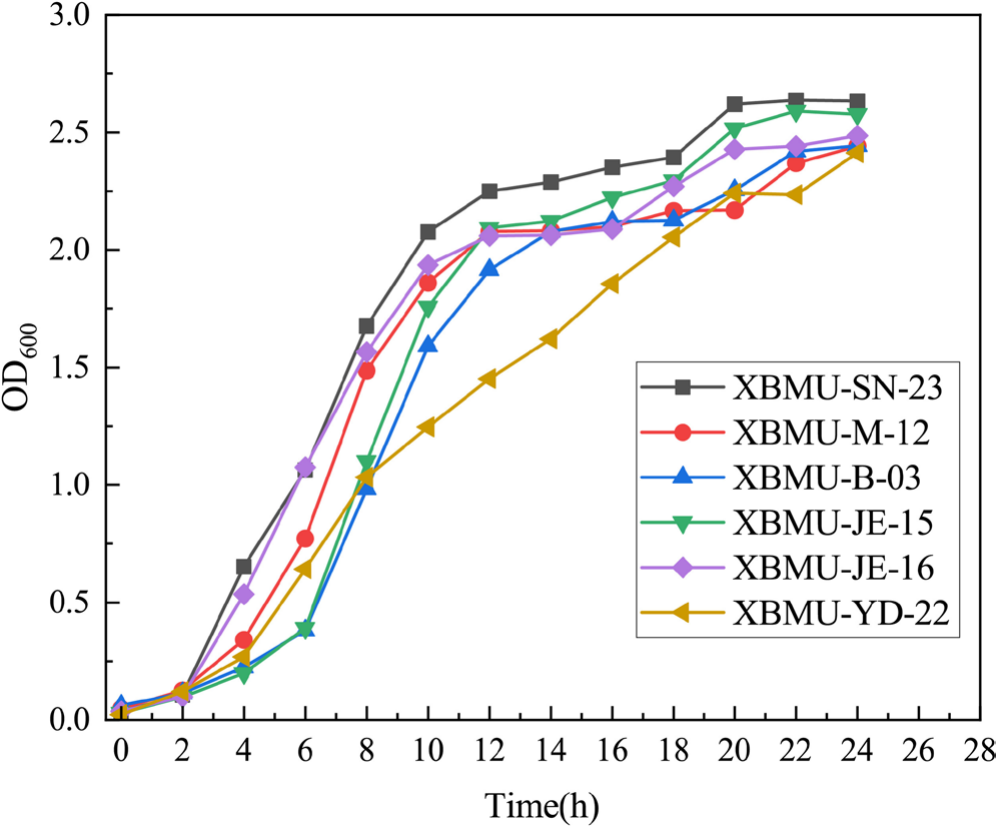
Growth capacity of GABA‐producing strains.

Figure 4 illustrates the growth trend of strain XBMU‐SN‐23, indicating that the overall growth activity of the strain is robust. From 0 to 10 h, the strain exhibits high growth activity with the most rapid growth rate. Between 10 and 18 h, it enters a stable phase, followed by a gradual increase, reaching its maximum optical density at approximately 22 h.

### 3.4 Acid Production Capacity of Strain XBMU‐SN‐23

**FIGURE 5:**
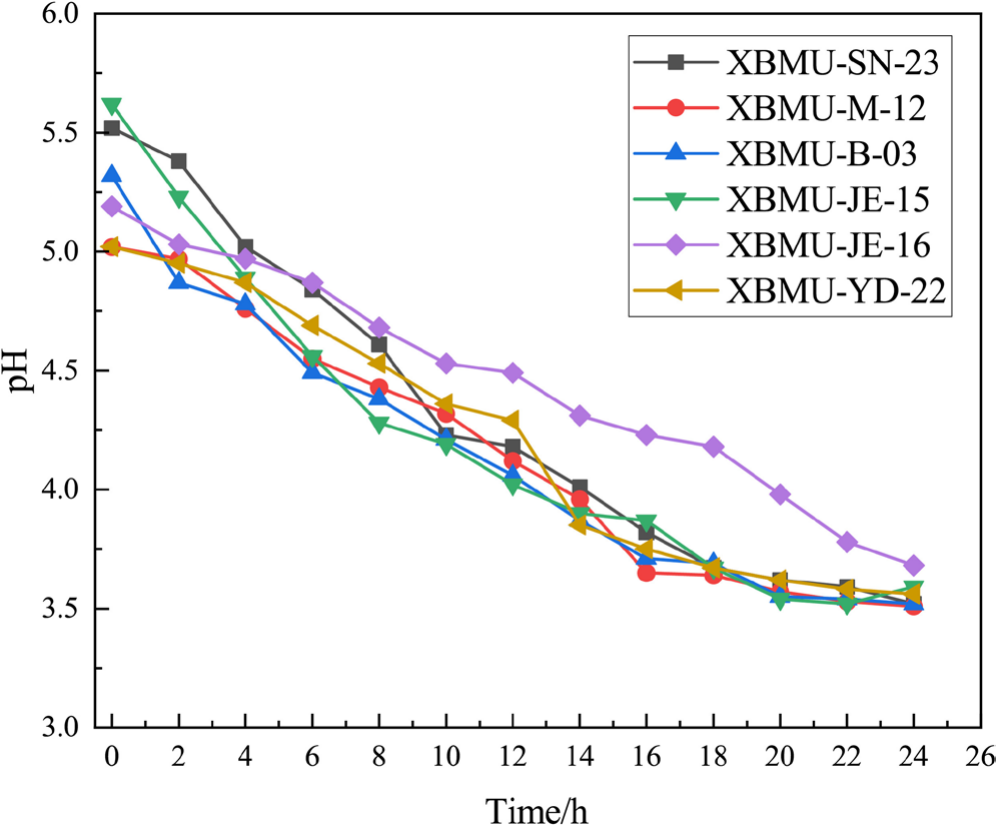
Acid production capacity of GABA‐producing strains.

As illustrated in Figure 5, strain XBMU‐SN‐23 produced acid rapidly during the first 10 h, resulting in significant pH changes. After 18 h, the strain entered a stabilization period, during which the pH remained stable. Strains exhibiting superior growth characteristics contributed to the optimization of the fermentation process, facilitating the identification of optimal medium composition and culture conditions to enhance product yield and fermentation efficiency.

### 3.5 Optimization of the Culture Medium Ingredients for Strain XBMU‐SN‐23

#### 3.5.1 Effect of Medium Components on GABA Yield by Strain XBMU‐SN‐23

**FIGURE 6:**
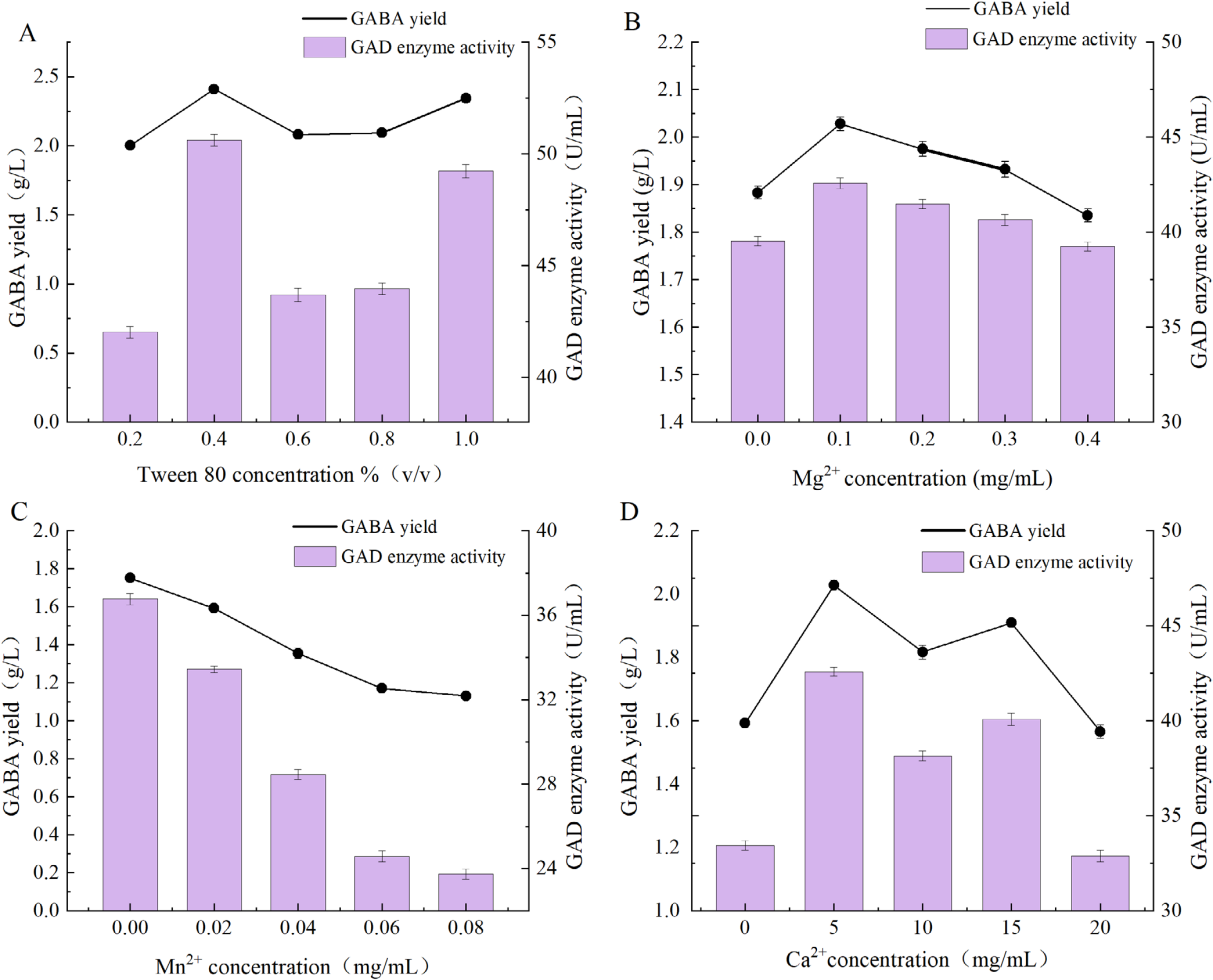
Effect of different medium component GABA production. (A) Tween 80 concentrations, (B) Mg^2+^ concentrations, (C) Mn^2+^ concentrations, and (D) Ca^2+^ concentrations.

As illustrated in Figure 6A, both GAD activity and GABA yield first increased and then decreased with the increase of Tween 80 concentration within the 0.2%–1.0% (v/v) concentration range. Fermentation broth supplemented with Tween 80 exhibited the highest GAD activity of 50.61 ± 0.016 U/mL and a GABA yield of 2.41 ± 0.029 g/L (*n* = 3) at the 0.4% (v/v) concentration.

The optimal concentration of Mg^2+^ at 0.1 mg/mL in the medium is illustrated in Figure 6B, where the GAD activity measured 42.61 ± 0.03 U/mL and the GABA yield was 2.03 ± 0.03 g/L (*n* = 3). Subsequently, as the concentration of Mg^2+^ increased, both GABA yield and GAD activity gradually declined. In Figure 6C, Mn^2+^ acts as a metal inhibitor, which reduced GABA synthesis and GAD activity. The addition of Mn^2+^ to the fermentation medium was minimal, resulting in the highest GABA yield of 1.75 ± 0.06 g/L and GAD activity of 36.76 ± 0.09 U/mL (*n* = 3) without the including of the Mn^2+^ component. It has been observed that the addition of Mn^2+^ can act as a metal accelerator, increasing the GAD activity of *Enterococcus avium* (Lim et al. 2022), or as a metal inhibitor, reducing the GAD activity of *Latilactobacillus sakei* OPK2‐59 (Yu and Oh 2011). Figure 6D shows that the optimal concentration of Ca^2+^ as a metal additive during fermentation was 5 mg/mL. At this concentration, the highest GAD activity of 42.58 ± 0.18 U/mL and a GABA yield of 2.027 ± 0.07 g/L (*n* = 3) were observed. GAD is the key rate‐limiting enzyme in the GABA biosynthetic pathway, and its activity is the main factor regulating GABA yield. An appropriate concentration of additives in the medium can significantly enhance the GAD activity of strain XBMU‐SN‐23, promoting the conversion of glutamate into GABA and thereby increasing GABA yield. In contrast, when the additive concentration exceeds the optimal range, GAD activity is markedly inhibited, resulting in a corresponding decrease in GABA yield. These results demonstrate a highly consistent correlation between GAD activity and GABA yield.

#### 3.5.2 Optimization of the Culture Medium Ingredients by RSM

The optimal culture medium components for GABA yield were determined using the OFAT method. Three factors were selected for RSM optimization: Tween 80 concentration (% v/v), Mg^2+^ (mg/mL), and Ca^2+^ (mg/mL), with GABA yield (g/L) as the response variable. Based on the *Box–Behnken* design, 17 experimental groups were designed, including five replicates at the central point. The results are presented in Table 4.

**TABLE 4:** *Box–Behnken* design consisting of 17 experiments used for the optimization of the culture medium.

Serial number	Factors			GABA yield (g/L)
	*X*_1_‐Tween80 (%v/v)	*X*_2_‐Mg^2+^ (mg/mL)	*X*_3_‐Ca^2+^ (mg/mL)	
1	0.2	0.0	5	1.945
2	0.6	0.0	5	2.083
3	0.2	0.2	5	2.234
4	0.6	0.2	5	1.871
5	0.2	0.1	0	2.125
6	0.6	0.1	0	2.026
7	0.2	0.1	10	2.321
8	0.6	0.1	10	2.175
9	0.4	0.0	0	2.325
10	0.4	0.2	0	2.267
11	0.4	0.0	10	2.245
12	0.4	0.2	10	2.422
13	0.4	0.1	5	2.542
14	0.4	0.1	5	2.554
15	0.4	0.1	5	2.533
16	0.4	0.1	5	2.618
17	0.4	0.1	5	2.564

By analyzing the multiple regression data in Table 4 with Design‐Expert v8.0.6.1 software, the quadratic multiple regression equation between GABA yield (*Y*) and Tween 80 concentration (*X*_1_), Mg^2+^ concentration (*X*_2_), and Ca^2+^ concentration (*X*_3_) was obtained as follows:

$$\begin{array}{c} Y=2.56-0.059X_{1}+0.025X_{2}+0.053X_{3}-0.13X_{1}X_{2}-0.012X_{1}X_{3}\\ \;+0.059X_{2}X_{3}-0.34{X_{1}}^{2}-0.19{X_{2}}^{2}-0.059{X_{3}}^{2} \end{array}$$

As shown in Table 5, the quadratic polynomial model was extremely significant (*p* < 0.01), and the lack of fit was not significant (*p* > 0.05), suggesting that the model had good fitting degree and validity. The quadratic equation adequately simulated the actual experimental effects of the three factors at three levels. The coefficient of determination *R* ^2^ = 0.9837 and adjusted *R* ^2^ = 0.9627 demonstrated a high degree of fit between the predicted and actual values, suggesting that the model was reliable and suitable for the optimization of medium additive concentrations.

**TABLE 5:** Anova and regression analysis of *Box–Behnken* design for GABA‐producing medium of *Lactiplantibacillus plantarum* XBMU‐SN‐23.

Source	Sum of squares	DF	Mean square	*F*	*p*
*X*_1_‐Tween80	0.028	1	0.028	13.98	0.0073**
*X*_2_‐Mg^2+^	0.048	1	0.048	2.43	0.0162*
*X*_3_‐Ca^2+^	0.022	1	0.022	11.16	0.0124*
*X*_1_ *X*_2_	0.063	1	0.063	31.77	0.0008**
*X*_1_ *X*_3_	0.00523	1	0.00523	0.28	0.6133
*X*_2_ *X*_3_	0.014	1	0.014	6.99	0.0332*
*X*_1_^2^	0.49	1	0.49	247.81	< 0.0001**
*X*_2_^2^	0.15	1	0.15	75.32	< 0.0001**
*X*_3_^2^	0.015	1	0.015	7.54	0.0287*
Model	0.83	9	0.093	26.72	< 0.0001**
Lack of fit	9.383 × 10^−3^	3	3.128 × 10^−3^	2.81	0.1715
Pure error	4.445 × 10^−3^	4	1.111 × 10^−3^		
Cor total	0.85	16	*R* ^2^ = 0.9837, *R* ^2^_Adj_ = 0.9627		

*Note: n* = 3 for each experimental run, three independent biological replicates.

* Represents *p*‐values where 0.01 < *p* ≤ 0.05.

** Represents *p*‐values where *p* ≤ 0.01.

**FIGURE 7:**
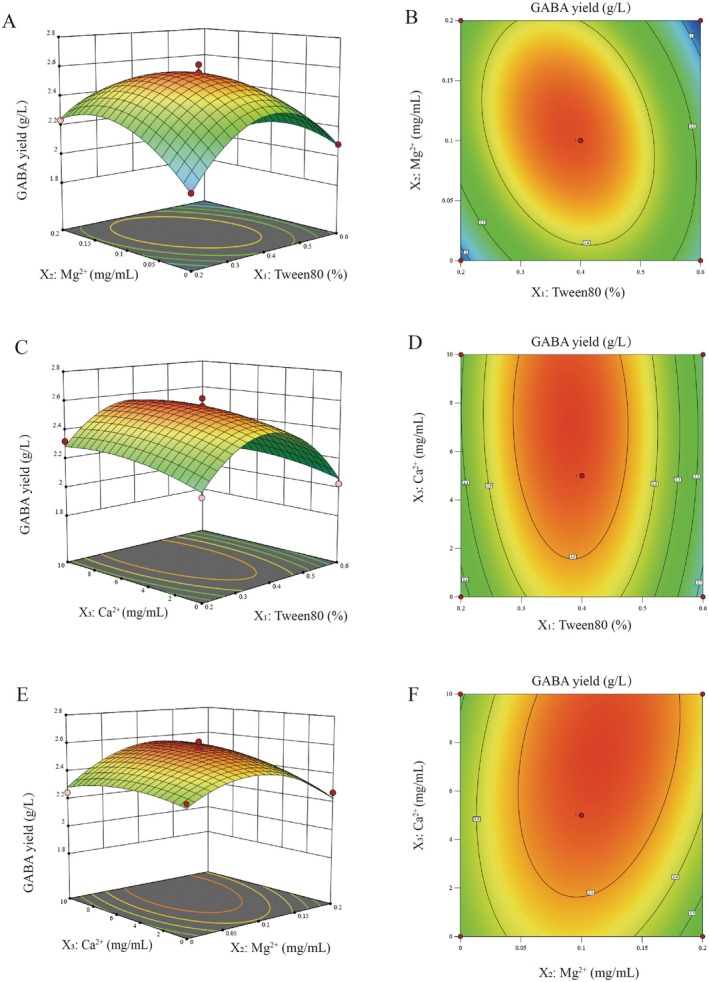
Response surface plot of the effects of different medium components on GABA yield. (A) 3D plot of the effects of Tween 80 and Mg^2+^ concentration on GABA yield, (B) contour plot of the effects of Tween 80 and Mg^2+^ concentration on GABA yield, (C) 3D plot of the effects of Tween 80 and Ca^2+^ concentration on GABA yield, (D) contour plot of the effects of Tween 80 and Ca^2+^ concentration on GABA yield, (E) 3D plot of the effects of Mg^2+^ and Ca^2+^ concentration on GABA yield, (F) contour plot of the effects of Mg^2+^ and Ca^2+^ concentration on GABA yield.

Three‐dimensional response surface plots were constructed to visualize the interactive effects of any two variables on GABA yield, with the third variable fixed at its optimal level. Figure 7A shows the 3D response surface plot for the interaction between Tween 80 and Mg^2+^. When Tween 80 concentration was fixed, GABA yield first increased and then decreased with rising Mg^2+^ concentration, forming a distinct peak, indicating a highly significant interaction between the two variables on GABA yield. The underlying mechanism is that Mg^2+^ serves as a crucial metal cofactor for GAD. It specifically binds to amino acid residues in the enzyme active site, stabilizes the native spatial conformation of the protein, reduces the activation energy required for the GAD‐catalyzed decarboxylation of l‐glutamate, and enhances the binding efficiency between the enzyme and its substrate, thereby improving GAD activity and GABA synthesis efficiency (Myung‐Ji et al. 2013; Laroute et al. 2021). Tween 80 can increase the cell membrane fluidity of the strain (Zaręba and Ziarno 2024) and promote the transmembrane uptake of Mg^2+^. The synergistic effect of these two components enhances the activation of GAD by Mg^2+^ more efficiently, resulting in the most significant interaction. Figure 7B shows the contour plot for the interactive effect of Tween 80 and Mg^2+^, which further verifies the significant synergistic effect of the two variables on GABA yield. The contour lines exhibit a distinct elliptical shape, with a clear central region and steep gradient, directly indicating a highly significant interaction between Tween 80 and Mg^2+^, which is completely consistent with the conclusion from the three‐dimensional response surface plot (Figure 7A). Figure 7C illustrates the interaction between Tween 80 and Ca^2+^. At a fixed Tween 80 concentration, GABA yield first increased and then decreased with increasing Ca^2+^ concentration, but the change was relatively gentle, indicating a weak interaction. Ca^2+^ can regulate ion channels on the cell membrane (Zhou et al. 2024) and promote the transmembrane transport of L‐glutamate from the fermentation broth, thus providing sufficient substrate for the GAD reaction. Since both Tween 80 and Ca^2+^ regulate cell membrane permeability with partially overlapping functions, their synergistic activation effect on GAD activity is thus relatively weak. Figure 7D presents the contour plot illustrating the interactive effect between Tween 80 and Ca^2+^, further corroborating the weak interactive effect of the two variables on GABA yield. Although the contour lines are elliptical in shape, their profiles are relatively gentle with a small magnitude of gradient change, directly reflecting that the interaction strength between Tween 80 and Ca^2+^ is far lower than that between Tween 80 and Mg^2+^. Figure 7E shows the interaction between Mg^2+^ and Ca^2+^. When the Mg^2+^ concentration was fixed, GABA yield first increased and then decreased with increasing Ca^2+^ concentration, indicating a significant interaction between the two ions. An optimal concentration of Ca^2+^ can indirectly enhance the activation of GAD by Mg^2+^ by improving substrate availability, thereby achieving synergistic regulation. However, excessive Ca^2+^ competes with Mg^2+^ for metal ion binding sites on GAD, antagonizing the essential activation effect of Mg^2+^, which leads to decreased GAD activity and inhibited GABA synthesis. Figure 7F shows the contour plot for the interaction between Mg^2+^ and Ca^2+^, further quantifying the significant interactive effect of these two metal ions on GABA yield. The contour lines exhibit a regular elliptical shape with a clear central high‐yield region and an obvious gradient change, directly confirming the significant interaction between Mg^2+^ and Ca^2+^ on GABA yield.

As essential additives in fermentation, metal ions directly affect GABA synthesis efficiency by regulating GAD activity. However, excessively high concentrations of these ions inhibit GAD activity in lactic acid bacteria. Analysis of the response surface data revealed that the significance of the interactions followed the order: Tween 80–Mg^2+^ (*X*_1_ *X*_2_) > Mg^2+^–Ca^2+^ (*X*_2_ *X*_3_) > Tween 80–Ca^2+^ (X_1_X_3_).

#### 3.5.3 Validation of the Optimal Conditions

Design‐Expert v8.0.6.1 analysis showed that the theoretical optimal medium conditions were 0.374% (v/v) Tween 80, 0.12 mg/mL Mg^2+^ and 7.755 mg/mL Ca^2+^, with a predicted theoretical GABA yield of 2.583 g/L (an increase of 20.14% compared with the initial yield of 2.150 g/L). For the convenience of practical industrial application, the optimal medium conditions were adjusted to the nearest practical values: 0.4% (v/v) Tween 80, 0.1 mg/mL Mg^2+^ and 5 mg/mL Ca^2+^. Three parallel validation experiments were conducted under the adjusted optimal medium conditions, and the actual GABA yield reached 2.618 g/L, which was slightly higher than the theoretical predicted value, indicating the reliability and practicality of the established regression model.

### 3.6 Optimization of the Fermentation Conditions for GABA Yield by Strain XBMU‐SN‐23

#### 3.6.1 Effect of the Culture Conditions on GABA Yield by Strain XBMU‐SN‐23

**FIGURE 8:**
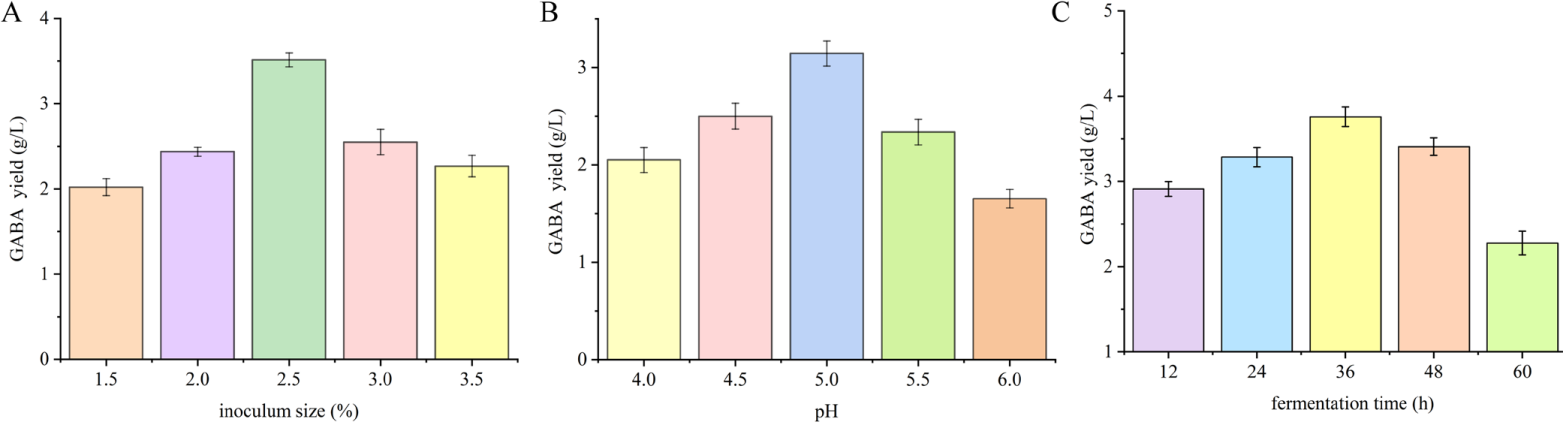
Effect of inoculum size, pH value, and fermentation time on the GABA of fermentation products (A–C).

As shown in Figure 8A, the yield of GABA by strain XBMU‐SN‐23 initially increased and subsequently decreased with the increase of inoculum size. The strain exhibited the highest GABA yield of 3.51 g/L at an inoculum concentration of 2.5% (v/v).

As illustrated in Figure 8B, the GABA yield increased at a pH range of 4.5 to 5.0, with the maximum yield of 3.14 g/L achieved at pH 5.0. Therefore, an initial pH of 5.0 was determined to be optimal for strain XBMU‐SN‐23. pH is a critical parameter for GABA yield, as the biosynthesis mechanism of GABA is closely associated with pH conditions (Liu et al. 2023).

Figure 8C illustrates that GABA yield increased from 12 to 36 h, reaching a maximum of 3.76 g/L at 36 h, and then decreased with the extension of fermentation time. Therefore, the optimal fermentation duration for strain XBMU‐SN‐23 was determined to be 36 h.

#### 3.6.2 Optimization of the Fermentation Conditions by RSM

The RSM is a three‐dimensional spatial surface that illustrates the results obtained from the interaction of various test factors. Three factors: pH, fermentation time (h), and inoculum size (%) were selected for optimization through response surface analysis tests. Seventeen experimental groups, including five replicated groups, were designed according to the *Box–Behnken* design, and the results are presented in Table 6. By analyzing the data from the multiple fitting regression tests presented utilizing Design‐Expert v8.0.6.1 software, the quadratic multiple regression equation for GABA yield (*y*) in relation to pH (*x*_1_), inoculum size (*x*_2_), and fermentation time (*x*_3_) was obtained as follows:

$$\begin{array}{c} y=3.41-0.14x_{1}+0.34x_{2}+0.34x_{3}-0.064x_{1}x_{2}-0.17x_{1}x_{3}\\ \;-0.016x_{2}x_{3}-0.27{x_{1}}^{2}-0.26{x_{2}}^{2}-0.42{x_{3}}^{2} \end{array}$$

**TABLE 6:** *Box–Behnken* design consisting of 17 experiments used for the optimization of the culture conditions.

Test number	Factors			GABA yield (g/L)
	*x*_1_‐pH	*x*_2_‐inoculum size (%)	*x*_3_‐fermentation time (h)	
1	4.5	2.0	36	2.683
2	5.5	2.0	36	2.533
3	4.5	3.0	36	3.352
4	5.5	3.0	36	2.946
5	4.5	2.5	24	2.318
6	5.5	2.5	24	2.351
7	4.5	2.5	48	3.413
8	5.5	2.5	48	2.782
9	5.0	2.0	24	2.009
10	5.0	3.0	24	2.864
11	5.0	2.0	48	2.635
12	5.0	3.0	48	3.425
13	5.0	2.5	36	3.432
14	5.0	2.5	36	3.562
15	5.0	2.5	36	3.453
16	5.0	2.5	36	3.312
17	5.0	2.5	36	3.283

As shown in Table 7, the quadratic polynomial model is statistically significant (*p* < 0.01), whereas the lack‐of‐fit term is not significant (*p* > 0.05), indicating a valid and well‐fitted model. The quadratic equation effectively simulates the actual experimental effects of three factors at three levels. Its coefficient of determination, *R* ^2^ = 0.9717, and adjusted *R* ^2^ = 0.9353 demonstrate a strong fit between the simulation and the actual data. This suggests that the experimental methodology employed in the model is both reliable and suitable for optimizing fermentation conditions.

**TABLE 7:** Anova and regression analysis of *Box–Behnken* design for GABA‐producing culture conditions of *Lactiplantibacillus plantarum* XBMU‐SN‐23.

Source of variance	Sum of squares	DF	Mean square	*F*	*p*
*x*_1_‐pH	0.17	1	0.17	11.06	0.0127*
*x*_2_‐ inoculum size	0.93	1	0.93	61.76	0.0001**
*x*_3_‐fermentation time	0.92	1	0.92	61.12	0.0001**
*x*_1_ *x*_2_	0.016	1	0.016	1.09	0.3315
*x*_1_ *x*_3_	0.11	1	0.11	7.32	0.0304*
*x*_2_ *x*_3_	0.00156	1	0.00156	0.070	0.7987
*x*_1_^2^	0.32	1	0.32	20.94	0.0026**
*x*_2_^2^	0.28	1	0.28	18.38	0.0036**
*x*_3_^2^	0.74	1	0.74	49.07	0.0002**
Model	3.62	9	0.4	26.72	0.0001**
Lack of fit	0.054	3	0.018	1.41	0.3622
Pure error	0.051	4	0.013		
Cor total	3.72	16	*R* ^2^ = 0.9717, *R* ^2^_Adj_ = 0.9353		

*Note: n* = 3 for each experimental run, three independent biological replicates.

* Represents *p*‐values where 0.01 < *p* ≤ 0.05.

** Represents *p*‐values where *p* ≤ 0.01.

**FIGURE 9:**
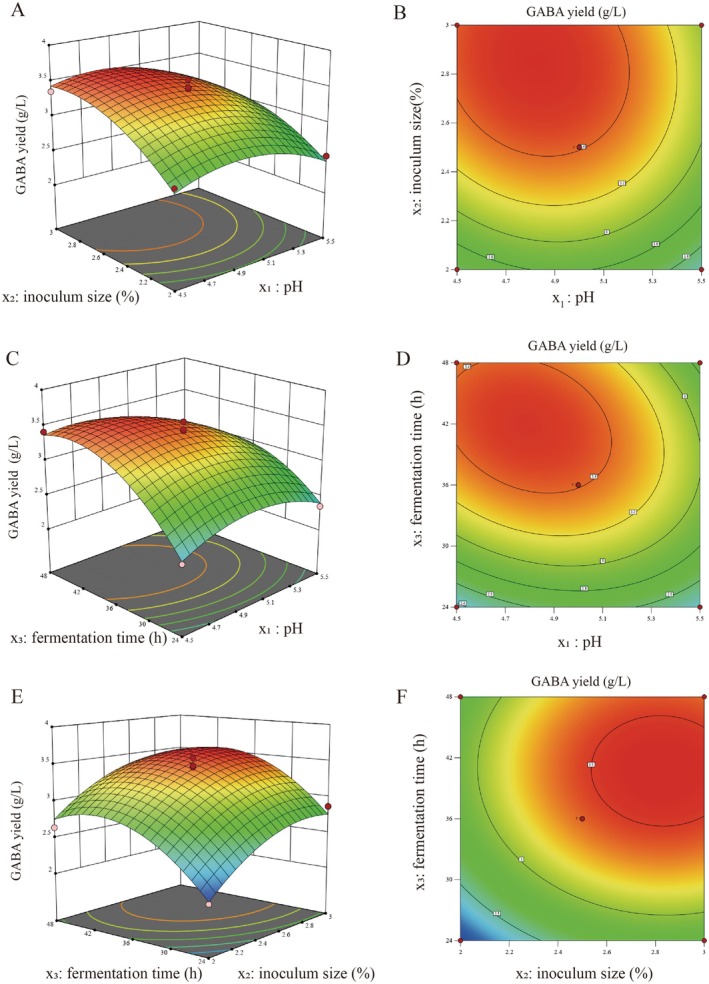
Response surface plot of the effects of different fermentation conditions on GABA yield. (A) 3D plot of the effects of pH and inoculation volume on GABA yield, (B) contour plot of the effects of pH and inoculation volume on GABA yield, (C) 3D plot of the effects of pH and fermentation time on GABA yield, (D) contour plot of the effects of pH and fermentation time on GABA yield, (E) 3D plot of the effects of inoculation volume and fermentation time on GABA yield, (F) contour plot of the effects of inoculation volume and fermentation time on GABA yield.

A three‐dimensional response surface plot combined with contour plot was generated using Design‐Expert v8.0.6.1 to evaluate the combined effects of independent variables on GABA yield, which is consistent with the standard interpretation logic of RSM. The interactive effect of pH and inoculum size on GABA yield is shown in Figure 9A,B. ANOVA showed that the interaction between these two factors was not significant (*p* = 0.3315), which was consistent with the nearly circular contour in the contour plot. As depicted in the 3D surface plot (Figure 9A), GABA yield exhibited a significant quadratic variation trend with changes in pH and inoculum size. The surface reached a peak at pH 4.5–5.0 and inoculum size 2.0%–2.5%, and thereafter, GABA yield decreased regularly as either factor deviated from this optimal range. These results demonstrate that this combination represents the optimal level interval for pH and inoculum size, and the synthesis of GABA is dominated by the main effects of these two single factors. In contrast, the interaction between pH and fermentation time was significant (*p* = 0.0304), corresponding to the distinctly elliptical contours and steep 3D response surface in Figure 9C,D. GABA yield reached the maximum value when the fermentation time was 36–42 h and the pH was maintained at 4.5–5.0. Fixing the pH at its optimal value, insufficient or excessive fermentation time both led to a significant reduction in GABA yield; conversely, fixing the fermentation time, deviation of pH from the suitable range also drastically impaired GABA synthesis efficiency. These findings confirm the synergistic regulatory effect between pH and fermentation time, highlighting the necessity of considering their matching relationship synchronously during process optimization. The analysis of the interactive effect of fermentation time and inoculum size (Figure 9E,F) showed that the interaction term was not significant (*p* = 0.7987) according to ANOVA, and this result was further verified by the circular contour profile in the contour plot. The 3D surface plot presented a typical unimodal characteristic, with GABA yield peaking at a fermentation time of 36–42 h and an inoculum size of 2.0%–2.5%. The variation trend of the surface was primarily driven by the extremely significant quadratic terms of these two factors (*p* < 0.01), indicating that their effects on GABA yield are relatively independent. Outside the optimal level interval, either excessively high/low inoculum size or insufficient/excessive fermentation time significantly inhibited the GABA synthesis capacity of *L. plantarum* XBMU‐SN‐23. The morphological characteristics of the 3D response surface plots and contour plots are highly consistent with the judgment of interaction significance from the ANOVA. The interaction between pH and fermentation time is the only significant interaction term, while the interactions between pH and inoculum size, as well as between inoculum size and fermentation time, are not significant. This order of interaction significance was mutually verified by statistical analysis and visual observation of the response surface.

#### 3.6.3 Validation of the Optimal Conditions

Based on the RSM analysis of fermentation conditions, the theoretical optimal fermentation parameters were predicted as initial pH 4.949, 2.806% (v/v) inoculum size and 40.196 h fermentation time, with a predicted theoretical GABA yield of 3.607 g/L under the pre‐optimized medium conditions. For practical application, the fermentation conditions were adjusted to the nearest practical values: initial pH 5, 2.5% (v/v) inoculum size and 36 h fermentation time. Three parallel validation experiments were carried out under the combined optimal medium and fermentation conditions, and the actual GABA yield reached 3.562 g/L, which was close to the theoretical predicted value and represented a 65.7% increase compared with the initial unoptimized yield (2.150 g/L).

## 4 Discussion

LAB are important microbial cell factories for GABA biosynthesis because they are generally recognized as safe (GRAS). GABA exhibits multiple physiological functions including improving sleep and alleviating anxiety, showing great application potential in various fields (Hou et al. 2024; Milon et al. 2024). Moreover, most GABA‐producing strains screened from fermented foods lay a solid foundation for their applications in the food industry. For instance, strain Y8 isolated from kimchi showed a remarkable increase in GABA yield along with fermentation time (Yu et al. 2017), and strain LS12‐1 from Thai fermented foods produced GABA up to 22.94 g/L (Phuengjayaem et al. 2021). Notably, the strain isolated in this study achieved efficient GABA yield without exogenous PLP, reaching a GABA yield of 2.150 g/L. In contrast, most reported high‐yield *L. plantarum* strains such as strain FRT7 require external PLP to maintain GAD activity (Cai et al. 2023). This feature suggests that the strain in this study may possess a complete endogenous PLP synthesis pathway, which can significantly reduce costs for industrial production.

RSM is a statistical technique combining experimental design and mathematical modeling for optimizing multifactor processes (Zewide et al. 2025). It establishes functional relationships between independent variables and response values, enabling systematic process optimization with fewer experiments, efficient evaluation of interactive effects, and reliable identification of optimal conditions. Therefore, *L. plantarum* XBMU‐SN‐23 was used in this study to optimize fermentation parameters for enhanced GABA yield. The GABA‐producing capacity of LAB is strongly affected by culture conditions, among which medium composition plays a crucial role. Microbial growth relies on specific nutrients, and changes in medium components alter nutrient availability, which in turn affects the metabolic activity and GABA synthesis capacity of LAB (Falah et al. 2021; Thongruck and Maneerat 2023). In this study, supplementation of the medium with 0.374% (v/v) Tween 80, 0.12 mg/mL Mg^2+^, and 7.755 mg/mL Ca^2+^ increased the GABA yield of *L. plantarum* XBMU‐SN‐23 to 2.583 g/L. Similar results have been reported previously. For example, Jiang et al. optimized the medium and fermentation conditions of *L. plantarum* Lp3 using RSM and obtained a maximum GABA yield of 3.74 g/L (Jiang et al. 2021). and the advantage of XBMU‐SN‐23 is that it does not require exogenous PLP addition, which has more industrial application potential.

GABA biosynthesis in LAB mainly depends on the catalytic activity of GAD, with PLP as its key coenzyme. Most high‐GABA‐producing *L. plantarum* strains reported to date require exogenous PLP to sustain high GAD activity (Icer et al. 2024). In this study, strain XBMU‐SN‐23 efficiently produced GABA without any exogenous PLP, indicating a strong innate PLP biosynthesis ability that is beneficial for industrial applications. GAD activity and GABA yield are coordinately regulated by metal ions, pH, and cell membrane permeability. Mg^2+^ acted as an essential metal cofactor for GAD and significantly stimulated GABA yield, while Ca^2+^ indirectly promoted GABA synthesis by improving substrate transport. In contrast, Mn^2+^ exhibited an inhibitory effect on GAD activity in this strain, revealing strain‐specific regulation patterns among different LAB. The optimal pH for GABA yield by XBMU‐SN‐23 was 5.0, and excessively low pH strongly inhibited GABA accumulation. Tween 80 improved cell membrane permeability and facilitated the transport of metal ions and substrates, showing a strong synergistic effect with Mg^2+^, which explains the highly significant interaction between these two factors. As a strain isolated from Lanzhou Jiangshui, XBMU‐SN‐23 exhibits natural acid tolerance. During fermentation, acid stress induces GABA synthesis, and accumulated GABA in turn alleviates intracellular acid stress, forming a self‐regulating metabolic balance. Culture conditions also significantly influence GABA yield in lactobacilli (Li et al. 2010). Tajabadi et al. reported that culture temperature is the most critical factor, and the optimal conditions for *L. plantarum* Taj‐Apis362 were initial glutamate concentration of 497.97 mM, temperature of 36°C, initial pH of 5.31, and incubation time of 60 h, with a GABA yield of 0.737 g/L (Tajabadi et al. 2015).

In this study, the highest GABA yield of 3.51 g/L was obtained at an inoculum size of 2.5%, and further increases in inoculum led to a gradual decrease in GABA yield (Figure 8A). As shown in Figure 8B, the lowest GABA content was observed at initial pH 4.0, while pH 4.0–5.0 was favorable for GABA synthesis. Thuy et al. reported the highest GABA yield by *Pediococcus pentosaceus* MN12 at pH 7.0 (Thuy et al. 2021), whereas Jiang et al. found that GABA yield by LAB increased significantly within pH 3.0–5.0 and peaked at pH 5.0 (Jiang et al. 2021). pH is a critical parameter because the biosynthetic mechanism of GABA is closely related to environmental pH (Pannerchelvan et al. 2023). Strain XBMU‐SN‐23 reached the maximum GABA yield of 3.76 g/L at 36 h of fermentation (Figure 8C). Prolonged fermentation led to severe nutrient depletion and environmental deterioration, resulting in the entry of the bacterial culture into the decline phase and the inhibition of GABA synthesis. For further optimization, RSM was applied to determine the optimal fermentation conditions: pH 5, inoculum size 2.5%, and fermentation time 36 h. Under these conditions, the actual GABA yield was 3.562 g/L, representing a notable improvement compared with previously reported values for *Lactobacillus* spp. (Shan et al. 2015). This study also has certain limitations that need to be addressed in future research. First, regarding strain identification, although 16S rDNA sequencing showed that strain XBMU‐SN‐23 shared 99% similarity with *L. plantarum*, this method has limited discriminatory power for closely related species within the *L. plantarum* group. Therefore, the current identification should be regarded as preliminary. More accurate species‐level confirmation can be achieved in the future using multilocus sequence analysis (MLSA) of housekeeping genes or whole‐genome sequencing. Second, the probiotic properties of the strain were not evaluated in this study. Subsequent studies should combine in vitro and in vivo experiments to comprehensively assess its suitability as a potential probiotic. Finally, in the subsequent medium and fermentation condition optimization experiments, the Berthelot colorimetric method was used for the rapid quantitative analysis of GABA, and HPLC was not used for parallel verification of all optimization samples. Although HPLC was used for the initial screening of high‐yield strains to ensure the accuracy of the primary results, the lack of HPLC parallel verification in the optimization stage may lead to minor errors in the quantitative results. In future large‐scale process optimization studies, HPLC will be used for quantitative analysis of all samples to ensure the absolute accuracy of the data. Despite the above limitations, the main objective of this study—isolating a high‐GABA‐producing strain and optimizing its fermentation conditions—has been successfully achieved. The conclusions provide a solid foundation for further in‐depth investigations.

## 5 Conclusion

In this study, a high GABA‐producing strain, XBMU‐SN‐23, was isolated from Jiangshui in Lanzhou and preliminarily identified as *L. plantarum* based on 16S rDNA sequencing. Taking GABA yield as the response value, the medium composition and fermentation conditions of *L. plantarum* XBMU‐SN‐23 were optimized by the combined method of OFAT and BBD‐RSM. Under the confirmed optimal culture conditions (0.4% Tween 80, 0.1 mg/mL Mg^2+^, 5 mg/mL Ca^2+^, initial pH 5, 2.5% inoculum size, 36 h fermentation time), the actual GABA yield of the strain reached 3.562 g/L, which was 65.7% higher than the initial unoptimized yield (2.150 g/L). This study clarifies the optimal fermentation conditions for high GABA yield of *L. plantarum* XBMU‐SN‐23 and provides a theoretical and experimental basis for the development of GABA‐enriched functional foods.

## Author Contributions

**Dandan Gao:** conceptualization, supervision, writing – review and editing, validation, funding acquisition. **Chenchen Wang:** methodology, data curation, investigation, visualization, writing – original draft, experimental implementation. **Jiwen Wang:** validation, experimental implementation. **Junyao Mei:** validation, experimental implementation. **Yujie Cao:** validation, experimental implementation. **Xuankang Yang:** validation, experimental implementation. **Jinpu Ma:** methodology, experimental implementation, data processing. **Shangyun Kang:** methodology, writing – review and editing, software, funding acquisition. **Jinyong Ma:** writing – review and editing, supervision, validation. **Ping Li:** writing – review and editing, validation.

## Funding

This study was supported by grants: the Fundamental Research Funds for the Central Universities of Northwest Minzu University (31920250026); the key projects of Gansu Province (25ZDCF001); the science and technology talent innovation project of Lanzhou (2025‐3‐023); the Science and Technology Program of Chengguan District, Lanzhou City (2025‐rc‐4); the key talent project of Gansu Province, (2024RCXM45); the National Key Research and Development Program of China (2024YFE0111400); and the National Natural Science Foundation of China (U24A20468).

## Ethics Statement

The authors have nothing to report.

## Conflicts of Interest

The authors declare no conflicts of interest.

## Data Availability

The data that support the findings of this study are available from the corresponding author upon reasonable request.
